# The complete mitochondrial genome of *Conaspidia wangi* Wei, 2015 (Hymenoptera: Tenthredinidae) and its phylogenetic analysis

**DOI:** 10.1080/23802359.2021.1944375

**Published:** 2021-07-05

**Authors:** Huilin Yang, Ziyun Lu, Meicai Wei, Gengyun Niu

**Affiliations:** College of Life Sciences, Jiangxi Normal University, Nanchang, China

**Keywords:** Mitogenome, gene rearrangement, Tenthredininae, phylogeny

## Abstract

The mitochondrial genome of *Conaspidia wangi* Wei, 2015 was described. The total length of the sequence was 15,924 bp. The overall A + T content was 80.4%. In comparison with the ancestral organization, *trnG* was reversed and translocated between the AT-rich region and *trnQ*, which was reported for the first time in Symphyta. The downstream gene order of the AT-rich region were thus arranged as *trnG*-*trnQ*-*trnM*-*trnI*.

*Conaspidia* Konow, 1898 is a peculiar genus of Tenthredininae and occurs in Eastern Asia (Wei and Nie 1997). The systematic position of the genus is uncertain till the present. Takeuchi (1952) placed *Conaspidia* into the tribe Sioblini s. lat. Wei and Nie (1998) placed it into tribe Sioblini s. str. Lacourt (1997) erected a tribe Conaspidini for the genus only under the subfamily Sioblinae, including Dimorphopterygini, Sioblini, and Athlophorini. In this study, we sequenced a mitochondrial genome of *Conaspidia wangi* and reconstructed a phylogenetic tree with other mitochondrial sequences of Symphytan species to clarify the phylogenetic position of *Conaspidia* within Tenthredinidae.

The specimen of *C. wangi* (CSCS-Hym-MC0100) was collected in Hujiashan, Zhenjiang Village, Jiangkou County, Guizhou Province, China (27.68°N, 108.82°E) in September 2018. The specimen was deposited at the Asia Sawfly Museum, Nanchang (ASMN) repository (Meicai Wei, weimc@126.com) under the voucher number CSCS-Hym-MC0100. Total DNA was extracted with DNeasyR Blood &Tissue Kits (Qiagen, Valencia, CA) and used according to the manufacturer’s instructions. Paired-end reads were sequenced using Illumina Hiseq 4000 platform at NOVO gene Ltd. (Tianjin, China). DNA sequences were assembled using MitoZ (Meng et al. 2019) and Geneious Prime (https://www.geneious.com). Annotations were generated in the MITOS web server (Bernt et al. 2013) and revised when necessary using Geneious Prime. The nucleotide sequences of 13 protein-coding genes (PCGs) of 34 species were aligned by TranslatorX (Abascal et al. 2010) and concatenated with Phylosuite (Zhang et al. 2020). The phylogenetic trees were constructed with GTR model in IQTREE Web Server (Trifinopoulos et al. 2016).

A total of 12,332,008,500 raw reads was obtained. One high-quality sequence yield by MitoZ was 15,832 bp in length and contained 37 genes. *Conaspidia punctata* (16,795 bp, unpublished) mitochondrial genome was utilized as a reference in Geneious Prime, with the mean depth of coverage across the sequences being 3648. Reassembly using *rrnS* and *trnG* as references extended contigs and obtained a 561 bp overlap. After manual verification, we obtained a control region of 789 bp in length. To verify the reliability of the results, we used the control region itself as a reference, and received a high-quality mapping with the flanking *rrnS* and *trnG* were found.

The complete mitogenome of *C. wangi* (MW415019) was 15,924 bp in length. In the mitogenome of *C. wangi*, there were 10 tRNAs (*trnG*, *trnQ*, *trnM*, *trnC*, *trnY*, *trnF*, *trnH*, *trnP*, *trnL*, *trnV*), two RNAs (*rrnL*, *rrnS*), four PCGs (*nad1*, *nad4*, *nad4L*, *nad5*) encoded on the N-strand, and the rest encoded on the J-strand (Figure 1). The A + T content was 80.4%, which was common in Tenthredinidae (Luo et al. 2019). There were six gene overlapping regions among *nad2*-*trnW* (2 bp), *cox1*-*trnY* (2 bp), *atp8*-*atp6* (7 bp), *atp6*-*cox3* (1 bp), *nad4*-*nad4L* (4 bp), and *nad6*-*cob* (1 bp). The *rrnL* and *rrnS* genes were 1368 bp and 804 bp in length, respectively. All PCGs initiated from typical ATN (ATG, ATT, and ATA) codon, except *cox1* with TTG codon. Among those PCGs, six initiated from ATG (*atp6*, *cob*, *nad1*, *nad2*, *cox2*, *cox3*), five initiated from ATT (*atp8*, *nad3*, *nad4L*, *nad5*, *nad6*), and one initiated from ATA (*nad4*) codon, respectively. All PCGs used TAA as a stop codon except for *nad1* (T). The gene order of *C. wangi* was consistent with that of *C. punctata*. In comparison with the ancestral organization (Boore 1999), the *trnG* was reversed and translocated between the AT-rich region and *trnQ*, which has never been reported previously in Symphyta. The downstream gene order of the AT-rich region was thus arranged as *trnG*-*trnQ*-*trnM*-*trnI*. The *trnQ*, *trnM*, and *trnI* clusters were only found in *Athalia* (He et al. 2019) and Megabelesisinae (Niu et al. 2021) within Tenthredinidae. In contrast, all other mitogenomes in this family have the interrupted corresponding tRNA-gene cluster.

**Figure 1.**
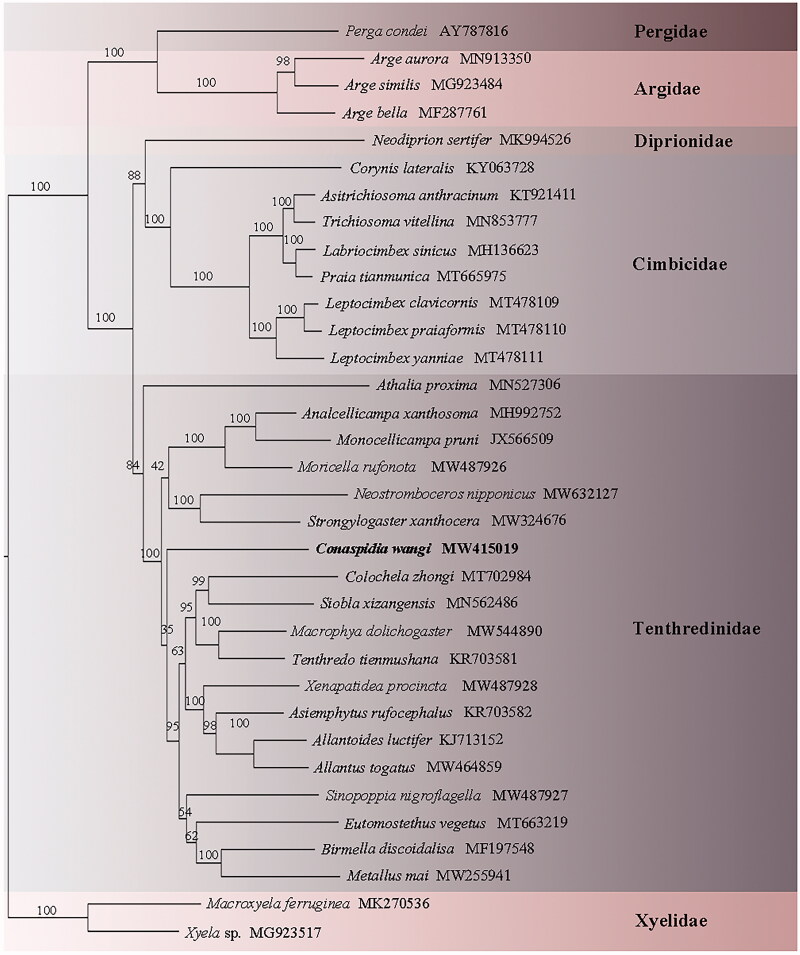
Maximum-likelihood tree based on the combination of thirteen PCGs of 34 Hymenopteran species. The accession number for each species is indicated after the Latin name.

Phylogenetic analysis was based on thirteen PCGs, with the third codon being removed. The result showed that *C. wangi* is a member of Tenthredinidae. However, it was not an ingroup of Tenthredininae but a sister group of (Tenthredininae + Allantinae) + (*Sinopoppia* + (Fenusinae + Blennocampinae)). *C. wangi* was remote from *Siobla xizangensis*; the latter is a member of Tenthredininae. More materials are needed to determine the natural position of *Conaspidia*. All related files have been uploaded to figshare (https://figshare.com/account/home#/projects/100028).

## Data Availability

The genome sequence data that support the findings of this study are openly available in GenBank of NCBI at [https://www.ncbi.nlm.nih.gov] (https://www.ncbi.nlm.nih.gov/) under the accession number MW415019. The associated BioProject, SRA, and BioSample numbers are PRJNA590371, SRR10490068, and SAMN13326122, respectively.
